# 104 Wildland Firefighters Suffer Increasing Risk of Job-Related Death

**DOI:** 10.1093/jbcr/irae036.103

**Published:** 2024-04-17

**Authors:** Kelsey G Glover, Steven A Kahn, Rohit Mittal

**Affiliations:** Medical University of South Carolina, Charleston, South Carolina; MUSC, Charleston, SC; MUSC/SJCH, Charleston, SC; Medical University of South Carolina, Charleston, South Carolina; MUSC, Charleston, SC; MUSC/SJCH, Charleston, SC; Medical University of South Carolina, Charleston, South Carolina; MUSC, Charleston, SC; MUSC/SJCH, Charleston, SC

## Abstract

**Introduction:**

Wildland firefighting is a niche specialization in the fire service - inherently dangerous with unique risks. Over the past decade, fatalities amongst all firefighters have decreased; however, wildland firefighter fatalities have increased. This subject has only been described in the grey literature, and a paucity of medical literature exists. With the recent wave of large-scale fire disasters across the US and Canada, the aim of this novel study is to analyze and better understand the trend of fatalities to eventually improve safety measures.

**Methods:**

The United States Fire Administration’s online fatality database was queried for on duty mortality between 1990 and 2022. The year 2001 was excluded due to the 340 deaths that occurred on September 11th. Data collected included demographics, incident characteristics, and nature of fatality. The data was then compared between the decades using a Fisher’s exact test.

**Results:**

From 1990-2000, there were 25 wildland fatalities out of 1,119 total firefighter fatalities, which increased to 75 wildland fatalities out of 1,190 total from 2002-2012. Between 2013-2022, there were 96 wildland fatalities out of 968, an increase from 2% to 10% of the total fatalities (p < 0.00001) from the first to last decade - a 500% relative increase. Despite the recent wave of 2023 wildfires across North America, the average annual number of wildfires has actually decreased 23% (from 79,829 to 61,377) between 1990-2000 and 2013-2022. Burn related fatalities have increased over time, from 9% of fatalities to 27% (p < 0.01), while trauma and motor vehicle crash fatalities have decreased significantly. MI accounted for 16% of total fatalities, with no significant change over time - however, number of years on the job was an independent risk factor for death from stress/exertion and MI, while age was not.

**Conclusions:**

Although wildfires have become less common over the past decade, there was a 5-fold increase in wildland firefighter fatalities. Burn related fatalities have also increased over time. Total number of years on the job increases the risk of death, while age does not, suggesting the job itself creates the risk of death - either from catastrophic incident or from cumulative inflammation from stress or byproducts of combustion. Further investigation, including analysis of 2023 data, is required to augment development of health and safety measures for these heroes. Multidisciplinary collaboration is necessary to mitigate the inherent job-related risk in this unique population.

**Applicability of Research to Practice:**

Burn centers are involved in caring for firefighters, thus must understand the relevant epidemiology and aid in risk mitigation for this population.

